# Occupational Risk Factors and Hypertensive Disorders in Pregnancy: A Systematic Review

**DOI:** 10.3390/ijerph18168277

**Published:** 2021-08-04

**Authors:** Emanuela Spadarella, Veruscka Leso, Luca Fontana, Angela Giordano, Ivo Iavicoli

**Affiliations:** Department of Public Health, Section of Occupational Medicine, University of Naples Federico II, Via Sergio Pansini 5, 80121 Naples, Italy; e.spadarella@gmail.com (E.S.); luca.fontana@unina.it (L.F.); giordanoa927@gmail.com (A.G.); ivo.iavicoli@unina.it (I.I.)

**Keywords:** maternal employment, preeclampsia, risk assessment and management, women at work, workplace conditions

## Abstract

Hypertensive disorders in pregnancy (HDP), including gestational hypertension (GH) and preeclampsia (PE), characterize a major cause of maternal and prenatal morbidity and mortality. In this systematic review, we tested the hypothesis that occupational factors would impact the risk for HDP in pregnant workers. MEDLINE, Scopus, and Web of Knowledge databases were searched for studies published between database inception and 1 April 2021. All observational studies enrolling > 10 pregnant workers and published in English were included. Un-experimental, non-occupational human studies were excluded. Evidence was synthesized according to the risk for HDP development in employed women, eventually exposed to chemical, physical, biological and organizational risk factors. The evidence quality was assessed through the Newcastle–Ottawa scale. Out of 745 records identified, 27 were eligible. No definite conclusions could be extrapolated for the majority of the examined risk factors, while more homogenous data supported positive associations between job-strain and HDP risk. Limitations due to the lack of suitable characterizations of workplace exposure (i.e., doses, length, co-exposures) and possible interplay with personal issues should be deeply addressed. This may be helpful to better assess occupational risks for pregnant women and plan adequate measures of control to protect their health and that of their children.

## 1. Introduction

Hypertensive disorders in pregnancy (HDP) are one of the most important problems in public health and perinatal medicine and include gestational hypertension (GH) and preeclampsia (PE) [1,2]. PE is a multisystem pregnancy disorder characterized by variable degrees of placental malperfusion, with release of soluble factors into the circulation that cause maternal vascular endothelial injury leading to hypertension and multi-organ injury [3]. It is defined as the development of hypertension after 20 weeks of pregnancy with proteinuria and/or evidence of maternal acute kidney injury, liver disfunction, neurological features, hemolysis or thrombocytopenia, or fetal growth restriction, and is one of the most common causes of maternal adverse outcome and death and a major contributor to perinatal morbidity and mortality [4]. PE is responsible for over 70,000 maternal deaths and 500,000 fetal deaths worldwide every year [5]. GH is intended as high blood pressure noted in the latter part of pregnancy, without other signs or symptoms of PE, and represents also a major cause of maternal, fetal, and newborn morbidity and mortality. A greater risk of abruptio placentae, cerebrovascular events, organ failure and disseminated intravascular coagulation has been reported in women with GH [6]. HDP complicate about 10% of pregnancies around the world, although their precise pathogenesis is unknown [7]. They are thought to be associated with an early abnormal placentation that may be caused by immunological, genetic, or environmental factors [8]. Genetic variants, including a single-nucleotide polymorphism in the angiotensinogen gene, have been reported among the nonmodifiable risk factors for HDP [9,10]. Maternal age, primiparous, multiple pregnancy, HDP in previous pregnancy, pre-existing hypertension, gestational diabetes mellitus (DM), type 2 DM, and urinary tract infections as well as a positive family history for such diseases and HDP have been also demonstrated as possible nonmodifiable risk factors [11]. Body mass index, anemia and a lower level of education, this latter aspect being related to a later start in prenatal care, have been included among modifiable risk factors [12].

The influence of environmental aspects on HDP, and possible underlying mechanisms of action, is not fully understood, although some evidence suggested that these factors may play a role in the etiology of such disorders [13]. The environmental temperature [14], and the exposure to air pollution [15,16] have been demonstrated as HDP contributing factors, while cigarette smoking [17,18] and calcium dietary intake [19,20] have been reported to play a protective role. Furthermore, epidemiological evidence strongly indicated that endocrine disruptors, such as bisphenol A and phthalates, could affect the physiological placental development, causing and/or contributing to the HDP onset [21,22].

In this context, although various adverse pregnancy outcomes, including low birthweight and preterm delivery have been reported in women who worked in adverse conditions or in jobs with known hazardous exposures [23], few studies have examined occupational risk factors for HDP. This seems an intriguing topic of research given the substantial HDP health burden and their increasing worldwide prevalence [24], as well as the ever-enhancing proportion of women who keep working outside home during pregnancy, maybe as a consequence of the increasing professional and financial demands that contemporary women face [25]. Data from the US National Survey of Family Growth found that approximately 60% of the US mothers worked ~6 months before their most recent delivery; 46% were employed in a full-time and 14% in a part-time schedule. Nearly 80% of these women continued to work during the third trimester of pregnancy [26].

Therefore, we systematically reviewed the effect of the employment status, and that of the exposure to a series of occupational risk factors, such as chemical, physical and biological risks, physical workload, as well as work organizational factors, including shift work and job-related stress, on the risk to develop HDP. Indeed, from the comparison between exposed and unexposed pregnant women with no history of HDP, we attempted to extrapolate evidence concerning the impact that participating in the workforce and being eventually exposed to the above-mentioned occupational risk factors during pregnancy may have on these specific adverse outcomes. From an occupational and public health perspective, this may be helpful to understand which may be the most hazardous work conditions for pregnant workers and which strategies should be primarily adopted to control risks and prevent adverse health effects for women and their children.

## 2. Materials and Methods

The Preferred Reporting Items for Systematic Reviews and Meta-Analyses Statement (PRISMA) criteria were followed to perform this systematic literature search [27]. We included observational studies (prospective cohort, retrospective cohort, and cross-sectional) and case series (with *n* > 10 enrolled pregnant women) that explored the risk for HDP development in pregnant women ≥ 18 years in relation to the employment status and the exposure to different occupational risk factors. To this latter aim, we considered eligible studies those enrolling pregnant workers exposed to chemical, physical and biological risks, physical workload, as well as work organizational factors, including shift work and job-related stress without restrictions of type and categorization of exposure as well as pregnancy periods involved. Exclusion criteria regarded reviews, case series (with *n*  <  10 enrolled women), conference papers, unpublished manuscripts, experimental studies on cellular and animal models, publications exploring HDP in not working women, studies that did not consider employment or the above-mentioned conditions of exposure as a variable, as well as all the papers published in languages other than English.

For this systematic review, three principal scientific databases: MEDLINE, Scopus, ISI Web of Knowledge and forward and backward citations were searched for studies published between database inception and 1 April 2021. We developed database-specific search strategies including a combination of subject headings (MeSH or Emtree) and keywords. The following key search terms were used in strategies specific to each database: (“preeclampsia” OR “gestational hypertension” OR “pregnancy induced hypertension” OR “pregnancy outcome”) AND (“maternal employment” OR “shift work” OR “occupational risk factor” OR “chemical exposure”) AND (“job stress” OR “job strain” OR “occupational health”). Two researchers, E.S. and V.L., independently reviewed titles and abstracts of all the 745 identified articles and discussed inconsistencies until consensus was obtained. Then, the two researchers independently screened the full text articles for inclusion. Additionally, in this phase, in case of disagreement, consensus on inclusion and exclusion was reached by discussion and, if necessary, a third researcher, I.I., was consulted.

Key information about the included studies was collected in a standardized data extraction form independently by two of the authors, E.S. and V.L., and extracted data were then compared in order to exclude any possible inaccuracy during the process. In detail, every outcome regarding an increased or decreased risk for HDP—reported as odds ratio (OR), adjusted OR (aOR), relative risk (RR), adjusted RR (aRR) and *p* for trend—was reported. Every included article was searched for details in location of the study and periods of the investigation, study design, characteristics of participants (number of cases and controls, where applicable), occupational risk factors explored with some details concerning the exposure features, results on the risk for HDP development.

**Figure 1:**
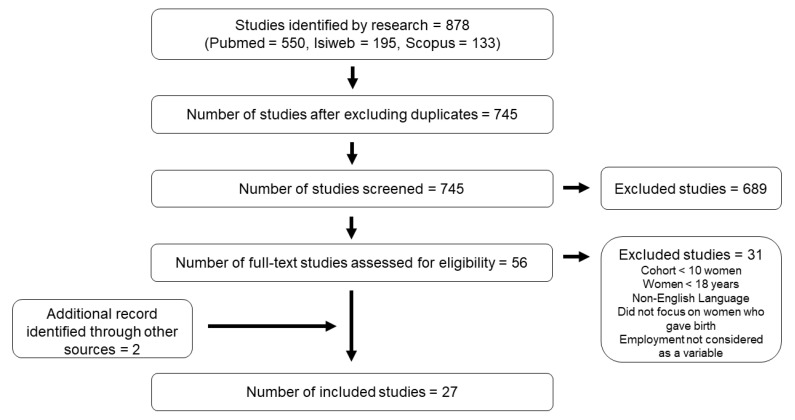
Flow diagram of literature research.

A consistent number of off-topic papers was included in our initial research due to the employment of both generic and specific keywords (Figure 1). These were mostly focused on environmental exposures rather than on occupational ones. Therefore, a high number of studies could be excluded via abstract consultation. The analysis of the reference lists accompanying published articles, performed to identify other relevant studies, allowed the inclusion of two additional eligible articles.

Quality of the selected studies was evaluated independently by two of the authors, E.S. and V.L., using the Newcastle–Ottawa quality assessment scale [28]. When the authors disagreed on the evaluation, the remaining authors also reviewed the article, and the judgement made by the majority of the reviewers determined the quality rating. Based on a maximum of nine points attributable within three different sections (Selection, Comparability and Outcome), a range scale was adopted, going from a sufficient evaluation with 6 points, a good evaluation for 7–8 points and an excellent evaluation for 9 points, the final evaluation was decided via discussion. Only one study was evaluated with less than 6 points, and we decided still to include it in this review. Possible biases concerning the design, conduct and analyses of the eligible studies were considered and addressed in the results and in the discussion section.

## 3. Results

A total of 27 studies were retrieved for the review purposes. We initially analyzed the association between work during pregnancy and HDP and then explored the possible impact of specific occupational risk factors on such disorders.

### 3.1. Work during Pregnancy and HDP

Despite the interest in understanding the association between HDP and the employment status during pregnancy, there were only few studies that addressed the relation of HDP with the raw variable “work” (Table 1). A first investigation by Landsbergis et al. [29], found a 5.1% cumulative incidence of HDP in employed women, while no cases of HDP were determined in non-employed ones (95% CI lower bound 1.1). In addition, Klonoff-Cohen et al. [30] found a significantly increased risk of PE in working versus non-working women (OR 2.3, 95% CI 1.2–4.6). The same results were later confirmed by Higgins et al. [31] in a prospective cohort of 993 normotensive women (aOR 5.48, 95% CI 1.08–27.76). A non-significant higher risk of HDP in female workers who left their jobs before the 34th week (OR 1.81, 95% CI 1.04–3.14) was demonstrated by Nugteren et al. [32], while Spracklen et al. [33] found a positive association with HDP when working hours per week were higher than 40 (aOR 1.72, 95% CI 1.00–2.98). Conversely, Eiríksdóttir et al. [34], showed that a higher risk of GH was determined in unemployed women in Iceland compared to employed ones (aOR 1.08, 95% CI 1.04–1.13), although no association could be found with PE.

Other investigations failed to confirm such positive or negative relationships with the employment status. In particular, in the study by Haelterman et al. [35] the prevalence of HDP in a cohort of working women was comparable to that observed in the general population (2.2% vs. 2.16). Similarly, no significant differences in HDP were detected in a cohort of 2422 women with respect to their employed or unemployed status [36]. However, caution should be applied in the interpretation of these preliminary data, as no information about the type of job and specific tasks performed was provided. Only few studies, in fact, included these aspects in their analyses. Chang et al. [37], in a cohort of 20,276 women found no significant differences between the group of employed vs. unemployed women (OR 0.98, 95% CI 0.78–1.24), although personnel engaged in the industrial sector was more represented in the first group. Bilhartz et al. [38] demonstrated that unemployed women had a significantly higher risk of HDP than homemakers (aOR 1.31, 95% CI 1.22–1.41). With respect to this latter group, also women engaged in business, management, teaching and healthcare professions, as well as in legal and social services had a significantly higher HDP risk [38]. Spinillo et al. [39] and Cerón-Mireles et al. [40] demonstrated that a retail job, involving the interaction with people to provide customer service and information about products and services, as well as handling and resolving complaints, was associated with a significantly higher risk of HDP (aOR 3.2, 95% CI 1.19–9.14 and aOR 2.8, 95% CI 1.6–4.7, respectively). In both studies, no association with HDP was found in industry and clerical jobs, these latter demonstrating a protective role with respect to PE development.

### 3.2. Occupational Risk Factors and HDP

To understand the impact that specific occupational risk factors may have on adverse pregnancy outcomes is absolutely important to plan suitable strategies to protect the health of pregnant women at work. The following paragraphs will detail the current available data on the relationship between biological, chemical, physical risk factors, including noise, vibration, as well as workload and organizational risk factors, such as shift works and job-related stress, with respect to HDP.

#### 3.2.1. Biological Risk Factors

Nowadays it is well established that the exposure to biological agents, e.g., *Chlamydia pneumoniae*, *Helicobacter pylori*, Cytomegalovirus, Human Immunodeficiency Virus, can be associated with PE, due to the consequent inflammatory response and the enhancement of uteroplacental acute atherosis [41,42,43,44]. However, although subjects experiencing occupational biological risks are normally provided with specific training on adequate job procedures, and on the employment of personal protective equipment to avoid/reduce the probability of exposure, to understand the relationship between such risk factors and the HDP onset remains a critical maternal health issue. Unfortunately, the current lack of available studies in this regard, prevents us from reaching conclusions. Moreover, in this perspective, information on the possible impact of common infections, like cold or stomach flu, in potentially exposed workers, like daycare staff and teachers, should deserve deeper attention to extrapolate data that may be useful to assess and manage biological risk in different occupational settings.

#### 3.2.2. Chemical Risk Factors

Few studies addressed the relation between occupational chemical exposure and HDP development (Table 2). When the association between the workplace exposure to organic solvents and the PE and GH manifestation was investigated in a cohort of 90 female workers, a significantly higher risk of PE was determined in exposed women compared to 180 unexposed controls (6.7% vs. 1.7%, *p* = 0.03; aRR 3.9, 95% CI 2.5–5.4, respectively) [45]. Of the women exposed to solvents who developed GH or PE, 8 out of 13 (62%) were exposed to aromatic compounds, 4 (31%) to halogenated solvents, and 1 (8%) to aldehydes. A higher incidence of GH was determined in women with an occupational exposure to organophosphorus pesticides compared to unexposed women (12% vs. 4%), with diazinon and malathion as the most likely causing agents (aOR 1.09, 95% CI 1.03–1.16 and aOR 1.14, 95% CI 1.08–1.19, respectively) [46]. No statistical association was evidenced by Nugteren et al. [32] between HDP and the exposure to several chemical compounds, i.e., polycyclic aromatic hydrocarbons, pesticides, phthalates, organic solvents, alkylphenolic compounds, metals, any chemical, as assessed via a mid (30 weeks) pregnancy questionnaire. Similarly, no association was found between the use of chemical agents and the onset of HDP by Irwin et al. [47], irrespective of the different levels of exposure considered.

#### 3.2.3. Physical Risk Factors

Although chronic noise has been associated with adverse pathophysiological effects, that may contribute to the progression of hypertension [48,49], the limited number of studies on occupational noise and HDP makes it difficult to extrapolate definite results on this issue (Table 3). Lissåker et al. [50] demonstrated a higher risk of HDP and PE for noise levels between 80 and 85 dB(A) in a nation-wide cohort, notably for first-time pregnant women who worked full-time, the subsample of the full cohort believed to be more likely to be truly exposed. In women in paid work beyond the 3rd month of pregnancy, Wergeland and Strand [51] found a slightly, although not significant, positive association between HDP and noise (OR 1.4, 95% CI 0.9–2.0, *p* = 0,1), even if no information on the levels of exposure was provided. Nurminen and Kurppa [52], in a cohort of mothers of children with congenital malformations, occupationally exposed to noise at a level around or greater than 80 dB(A) while performing manual occupations, found a significantly increased risk of GH in exposed compared to unexposed ones (RR 1.8, 95% CI 1.0–1.3). The relation between noise and GH was more evident in the shift work group, with the highest percentage (23%) of GH determined in the mothers with exposure to moderate or high intensity.

Conversely, in women employed since the first month of pregnancy in a noisy environment, intended as a place where the workers needed to speak loudly to be heard by someone at 2 m, no association between noise and HDP could be established [35]. Comparably, no relationship could be demonstrated when women’s job titles were classified as low, medium, and high noise exposure [47].

The effects of extreme temperatures or whole-body vibration (WBV) on HDP is a quite unstudied topic (Table 3). Haelterman et al. [35] and Irwin et al. [47] showed no association between extreme temperatures and HDP. Only two studies analyzed the association between exposure to WBV and HDP risk in women [35,53]. Haelterman et al. [35] found that WBV slightly raised, although not significantly, the risk of PE (aOR 1.2). More recently, Skröder et al. [53], derived WBV exposure from a job–exposure matrix and divided the levels of exposure into four categories (0, 0.1–0.2, 0.3–0.4 and ≥0.5 m/s^2^). The authors found a significant increased risk for both PE (aOR 1.76; 95% CI 1.41 to 2.20) and GH (aOR 1.55) in full time working women exposed to the highest WBV levels, while low (0.1–0.2 m/s^2^) or medium (0.3–0.4 m/s^2^) levels were not associated with a significantly increased risk of any outcome. The association with the ≥0.5 m/s^2^ WBV exposure was confirmed also when the samples were restricted to women with low education (PE: 1.78 aOR; GH: 1.59 aOR), first-time mothers (PE: 1,67 aOR; GH: aOR = 1.59) and to those without co-exposure to mechanical shocks (PE: 1.95 aOR; GH: 1.77 aOR) in comparison to unexposed controls. There were no clear associations for women working part time. Overall, these findings indicate that reassignment or pregnancy allowance is a necessary preventive measure for pregnant employees. In line with these statements, the European Agency for Safety and Health at Work (Eu-OSHA) emphasized the need to inform occupational physicians about women’s exposure to vibration and the need to take specific measures to limit women’s exposure and to prohibit it during pregnancy [54].

#### 3.2.4. Physical Workload

Conflicting results concerning the possible association between physical activity during work and HDP have been reported (Table 4). Some of the authors suggested a possible protective effect of physical activity on HDP [29,33,36]. Saftlas et al. [36], in fact, demonstrated that any regular physical activity at work conferred protection, although not significantly, against PE. Comparably, Landsbergis et al. [29], considering the physical work demand as the sum of climbing, exerting a lot of physical efforts, lifting, carrying, pulling, or pushing objects, reported a not significant protective effect. Spracklen et al. [33] found a significantly lower risk of PE when women spent more than 8.25 h active per day, including both the physical activity at work, at home and during leisure time.

Conversely, other authors reported a positive association between physically demanding work and HDP development. In Higgins et al. [31], women who had jobs classified as active (*n*. 49) had significantly higher mean daytime diastolic (*p* = 0.065), night time diastolic (*p* = 0.04) and 24 h diastolic (*p* = 0.02) blood pressure compared with women in jobs classified as sedentary (*n*. 135). Spinillo et al. [39] classified a population of severe PE affected women in four levels of job activity, i.e., no work, mild, moderate, and high physical activity, based on the type of work, physical intensity, posture at work, and weekly working hours. The authors found a significant linear trend relating the level of physical activity to the risk of PE, although the moderate/high physical activity at work was associated with a 2-fold increase in the risk of severe PE compared to mild activity (aOR 2.1, CI 1.18–3.75). When parity was considered as a possible classifying factor, nulliparas employed in jobs involving high levels of physical activity were at significantly decreased risk of GH compared to nulliparas working at low levels of physical activity (construction craftsmen, RR 0.37; unskilled laborers RR 0.71) [47]. Conversely, Nugteren et al. [32] found no association between HDP and physically demanding work-related risk factors, including long periods of standing, walking, or driving (more than 4 h/day), heavy lifting, and working hours.

According to specific types of physical demands, Wergeland and Strand [51], found a significantly positive association between PE and lifting heavy loads of 10–20 kg (aOR 1.8, 95% CI 1.0–2.0, *p* < 0.05). Irwin et al. [47] demonstrated a protective trend for PE in nulliparous women for jobs involving high level of lifting (RR 0.68, 95% CI 0.47–0.98), while reported a risk of twofold for PE when lifting occurred in parous women (RR 2.0, 95% CI 0.87–4.5). The study of Haelterman et al. [35] was the only one to assess the association between stairs climbing and PE. The comparison between 99 cases of PE versus 4381 normotensive controls pointed out a significant higher risk of PE in women climbing stairs frequently (aOR 2.3, 95% CI 1.2–4.1). Regarding standing for more than one hour per working day at work and possible associations with HDP, non-homogeneous evidence is available. Women standing daily at least 1 h consecutively, without walking, experienced a higher risk of PE (aOR 2.5, 1.4–4.6) in Haelterman et al. [38]. Spinillo et al. [39] reported a significant association with PE, although they considered prolonged standing as a part of physical exercise (at multivariate analysis likelihood chi-square = 9.38, 3 df, *p* = 0.002). Spracklen et al. [33] detected a positive association between the amount of average time spent standing in one place and GH (aOR 1.11, 95% CI 0.99–1.24). Different studies [32,36,40,51], nevertheless, reported no significant association between standing for more than one hour and HDP. Irwin et al. [47] demonstrated that jobs requiring high levels of standing were associated with a reduced risk of GH in nulliparous (RR 0.87, CI 0.69–1.1) and with an increased risk in parous women (RR 1.5, CI 0.95–2.5), respectively.

The association between specific movements reiteration per day and HDP was investigated in a couple of studies [35,51]. Women pulling or pushing objects more than 5 times per working day had non-significant higher risk of PE or GH according to Haelterman et al. [35] (aOR 1.6, 95% CI 1.0–2.5). Wergeland and Strand [51] reported that bending and twisting were not associated with PE, while a positive association was found with spending time with arms kept above shoulder level (aOR 1.4, 95% CI 1.0–2.2), even though Haelterman et al. [35] failed to confirm such relationship.

#### 3.2.5. Shift and Night Shift Work

The International Labour Organization defined shift work as a “a method of organization of working time in which workers succeed one another at the workplace” [55]. According to the European Union, night work means “working at least 3 h of the daily shift or a certain proportion of the yearly working time in a period of 7 h defined by national law and including the time from midnight to 05:00” [56]. Several authors have suggested that the alterations in the circadian rhythm caused by shift and night shift work, could negatively influence the women’s reproductive health, having an impact on both obstetric, maternal and fetal outcomes [23,57].

Wergeland and Strand [51] firstly described a significant association between shift work and PE, though it was only restricted to parous women (OR 2.0, 95% CI 1.1–3.6) (Table 5). More recently, Hammer et al. [58] demonstrated that among women working night shifts during the first 20 weeks of pregnancy, the risk of HDP grew with increasing number of consecutive night shifts (1.41, 95% CI 1.01–1.98) and of quick returns after night shifts (OR 1.28, 95% CI 0.87–1.95). The risk was even more glaring when a subgroup of obese shift-workers with BMI > 30 kg/m^2^ who worked long night shifts, longer spells of consecutive night shifts, and had the highest number of quick returns after nights, was analyzed (aOR 5.31, 95% CI 1.03–1.40). However, a possible bias, represented by the type of occupation performed, and the co-exposure to additional risk factors should be carefully considered for a correct interpretation of the results. In fact, night workers were mostly represented by nurses (61.3%) and physicians (17.9%), while these groups of workers were present in lower proportions, 14.2% and 8.8%, respectively, among women engaged in day works. A significantly increased aORs was observed for HDP in women working > 46 and 36–45 h per week with 1–5 night shifts per month during the second/third trimester of pregnancy (aOR: 2.02, 95% CI 1.39–2.93 and aOR 1.56, 95% CI 1.14–2.12, respectively) [59]. Interestingly, the increased risk, however, was not observed for those working night shifts ≥ 6 times per month. In these categories, the most representative occupation was nursing, potentially suggesting that better health management during pregnancy was available for this category of workers. Davari et al. [60] reported a significantly higher prevalence of PE in women engaged in shift works (14.5%) compared to morning works (7.9%), but the relation became non-consistent when the odds ratio was adjusted for parity and type of employment. Conversely, several studies described a lack of association between night shifts and HDP. In detail, Chang et al. [37] found no association between GH or PE and maternal shift work (aOR 0.96, 95% CI 0.63–1.46 and aOR 0.82, 95% CI 0.42–1.61), also when different job schedules were investigated, such as daytime only, evening only, daytime and evening only, rotating shift. Haelterman et al. [35] and Nugteren et al. [32], as well, reported no association with HDP. The first one analyzed women who had been employed since the first month of pregnancy, for at least 4 consecutive weeks of 20 h each, while the second one investigated women who started working before conception or during the first trimester. None of these studies took into account the type of employment. Nurminen [61] examining a cohort of mothers of children with congenital malformations who had worked temporarily or regularly in rotating shifts during pregnancy, found a significantly increased adjusted risk of GH among those exposed to noise in shift work compared to those in noiseless shift work (1.9 aOR, 95% CI 0.6–5.6). Without noise exposure, the mothers in shift work had experienced no more pregnancy-induced GH than the mothers with normal day work.

#### 3.2.6. Job Related Stress

Job related stress was investigated in regard to both hectic work pace and job strain (Table 6). Hectic work pace’s influence on the onset of HDP is controversial. Wergeland and Strand [51] found a significantly higher risk of PE in women engaged in hectic work pace beyond the 3^rd^ month of pregnancy compared to those with no hectic pace (OR 1.4, 95% CI 1.0–2.0). Such association was positively related to the frequency/length of such type of job rhythm, i.e., not daily, daily less than half time, daily more than half time. Additionally, the authors could determine a significant reduction in the risk of PE in women who had the possibility to influence their work pace compared to those who had not (OR, 0.7; 95% CI 0.5–1.0). Conversely, Haelterman et al. [35] found that women who never or rarely had a work break (OR 1.7, 95% CI 1.0–1.9) or those who had no control of the time of their breaks (OR 1.8, 95% CI 1.0–3.2), and those working with a forced pace (OR 1.6, 95% CI 0.8–3.1) tended to experience an increased risk of PE, although not always statistically significant. When job strain was assessed through the Karasek’s Job Demand-Control model [62], the adjusted odds ratio for PE was 1.7 (95% CI 0.8–3.3) for the women with high job strain (high demand–low latitude) with respect to those with low job strain as reference (low demand–high latitude). In contrast, the risk of GH was not increased by job strain. Using the same model, Marcoux et al. [63] demonstrated that a high job strain, intended as high demand–low decision latitude jobs, had a significant correlation with PE (aOR 2.1, 95% CI 1.1–4.1), with high psychological demand being the major cause (aOR 2.7, 95% CI 1.3–5.6), compared to conditions of low job strain, characterized by low demand–high decision latitude job. Klonoff-Cohen et al. [30] achieved comparable results. Additionally, comparing women in low strain jobs with non-working women, these authors found a higher risk of PE for the first ones (OR 2.0, 95% CI 1.4–4.3). Landsbergis et al. [29], conversely, could demonstrate a significant correlation with GH for low job complexity (aOR 2.1, 95% CI 1.0–4.6) and low job decision latitude (aOR 2.4, 95% CI 1.1–5.2), while no significant correlations were demonstrated for PE. On the other hand, Anorlu et al. [64] observed that stressful work during pregnancy (aOR 2.10;95% CI 1.20–3.71) was associated with increased risk of PE. The Work Experience and Appreciation Questionnaire of van Veldhoven and Meijman, was used to explore well-being, stress, psychosocial job demands, skill use, job control, social-organizational and employment conditions [65]. The authors examined the work-related stress on the basis of four variables: total working hours, defined as weekly hours of a paid work; workload, measured as pace work and both mental and physical workload; job control and job strain. No association between any variable analyzed and the development with PE and GH was detected.

Lastly, emotional stress was also associated with HDP, as stated by Leeners et al. [66]. Although, generally, major causes of emotional stress rely on not work-related situations, like divorce or health, emotional stress could be also determined by occupational problems, that, in turn, resulted significantly correlated with HDP.

## 4. Discussion

This review focused on a still challenging research issue concerning women at work and attempted to provide a comprehensive overview on the possible association between a variety of occupational risk factors and HDP. This seems an even more relevant topic, considering the currently enhanced number of women working during pregnancy, that is further expected to increase in the upcoming years [67]. However, despite this figure, our knowledge concerning the possible impact of working conditions on HDP development is still in a preliminary phase, maybe due to the relatively limited number of papers published on the topic in the past years that prevented to draw any definite conclusion.

Few studies, in fact, analyzed the possible correlation between a series of work-related variables that may influence HDP manifestation, such as job classification, working schedule, occupational risk factors and levels of exposure. This seems absolutely important to define possible “occupational targets” that should be adequately considered for the assessment of workplace risks able to induce adverse health effects on pregnant workers and the development of strategies to manage such dangerous job conditions.

As previously stated, employment classification can have a role in influencing the HDP development, i.e., retail jobs have been associated with a higher risk, while clerical ones appeared related with a lower one [40]. In [38], professional women were identified at higher risk for HDP than women employed as skilled laborers or working in the services sector. These associations were hypothesized to be due to the psychosocial and physical job stressors experienced in such business employment that could exceed the protective benefits associated with belonging to higher socioeconomic classes [38]. Conversely, other studies found no significant association between the type of employment and HDP [35,36,37].

The conflicting evidence that emerged from these revised studies further support the idea that, apart from the job title, a deeper analysis of a series of occupational aspects seems necessary, as they all could play a key role, possibly functioning in an additive or synergic mode, in determining different levels of HDP risks in various occupations. In this view, more and better designed studies are needed in order to confirm and extend this hypothesis with particular attention paid to the specific occupational risk factors experienced in each type of employment.

As to the frame of occupational risk factors, biological risks, despite being associated with the onset of HDP in extra-occupational contexts, have not been sufficiently explored in workplace settings. It is important to highlight that a significant percentage of the female workforce, mostly in healthcare field, can be exposed to a number of biological agents, despite the adoption of suitable work procedures and the employment of personal protective equipment aimed to control the exposure. Therefore, the impact of biological agents, like common infections, in women working during pregnancy, should be clarified in order to minimize risks and guarantee appropriate medical surveillance.

Regarding chemical risk factors, the few studies available in literature gave inconclusive results. Solvent-exposed women were approximately four times more likely to develop PE, compared to unexposed controls in the study performed by Eskenazi et al. [45] in 1988. However, some caution should be applied for a correct interpretation of these results, as no levels of exposure were provided, and no specific classes of solvents or single occupation could be demonstrated as more likely associated with PE. Additionally, the biological plausibility of the retrieved association between solvent [45], organophosphorous pesticides [46] and HDP should be verified according to the most recent advances on the toxicokinetics and toxicodynamics of the investigated substances, their possible mechanisms of action, also as endocrine disruptors, the improvements occurred in the workplace hygiene conditions and measures of control over time that significantly reduced the levels of exposure.

Overall, chemical risk should be strictly regulated in order to avoid/reduce exposure to all workers. Is quite harsh to assess the level of exposure in the subgroup of pregnant working women, since collective preventive measures are adopted to control the exposure in the workplaces, the use of personal protective equipment is mandatory, shifts are organized in order to minimize exposure and many recommendations are encouraged (e.g., wear work-only clothes once in the workplace, to maintain a good workplace hygiene), and finally, pregnant women are removed from chemical exposures as soon as they are aware about their status. The lack of an exposure–HDP association may be dependent on the effectiveness of the adopted preventive strategies, responsible for a reduction in the levels of exposure and the protection of workers. However, it is important to consider that these regulations are not always strictly followed and suitable interventions that facilitate compliance with good behaviors at work should be strongly pursued to decrease chemical exposures [67]. Additionally, possible conditions of individual susceptibility should be deeply addressed to define targeted protective measures.

Concerning physical risk factors, literature data did not provide conclusive information on the correlation of workplace noise exposure and HDP. However, a significant positive association was described with higher occupational noise levels between 80 and 85 dB(A) [50,52] particularly in co-exposure with night shift work [52,61]. As noise exposures in occupational settings are very different in their features, further research is necessary to define a possible dose–response relationship, specific molecular mechanisms of action and to deeply investigate the complex interplay between noise characteristics, in terms of i.e., type, intensity and duration of exposure, and individual characteristics of women, i.e., age of the pregnant women, anthropometric parameters and pregnancy periods of exposure, as well as collective and individual protective measures adopted in the workplace in influencing HDP onset [49,51]. No sufficient information is currently available on the role of extreme temperature and vibration exposure in determining HDP, with only one study showing a statistical association between exposure to WBV and the risk of HDP [53].

Despite the higher number of available studies, physical workload is still an incompletely understood risk factor for HDP. A possible explanation for the discordant results about the correlation between physical workload and HDP can derive from the complex and diverse physical demands required by the various job tasks. In fact, it seems that specific movements, such as climbing stairs, standing still, and lifting heavy objects, are associated with HDP, while other kinds of activities may have a weaker and not always confirmed positive relationship. In this view, physical activity, or at least time spent active per day during pregnancy, were reported to have a protective effect on the onset of HDP. However, some discordant results obtained for the whole physical workload might be interpreted as the sum of both harmful and protective effects of physical activity on pregnant workers. Additionally, it is important to consider possible co-exposure to other occupational risk factors, including organizational risks, the job-related stress and hectic pace of work, as a potential confounding issue. Moreover, self-reported data on occupational exposure should be interpreted with caution, as physical activity can be referred by women in many different ways depending on the subjective experience. Overall, pregnant women should be reassigned or have their tasks changed to reduce physical duties.

The association between shift work and HDP is controversial. Several studies confirmed the lack of this association following the first work of Wergeland and Strand [51] published in 1997. However, more recently, some authors could demonstrate a possible positive correlation, taking into account different confounding variables, such as BMI and parity [58,60]. It is widely ascertained that parity may differently affect HDP, as parous women experience GH, but not PE more frequently than nulliparous women [68]. Additionally, BMI seems to play an important role as a confounding factor due to the well-known correlation between night shift work and overweight and obesity [69]. This relation may have a mediating role in the shift work–HDP relationship. Therefore, it is important to consider these factors and adjust the selection of the population or the odds ratios accordingly. Long working hours, as part of the organization of the work schedule, should be also taken into consideration as a possible influencing factor for HDP development. This seems even more relevant taking into account that an inverse relationship has been reported between hours worked and leisure time, as women spending longer hours in work presumably have less leisure time activity [70].

Lastly, one possible confounder of those previously described associations might be job strain or emotional stress, that were not often taken into account in the analyses, despite the evidence for their influencing role in HDP. This aspect should be considered as a possible bias for the correct interpretation of the previously reported results. Moreover, work pace, as a possible component of job strain, was associated with the onset of PE, especially for women who could not have control over their occupational rhythm of activity. It must be pointed out that, due to legislations, in several nations women are already exempted from fatiguing physical exercise as stated before, or from being exposed to chemical substances or other risk factors. In this scenario, it may be important to have further investigation on the effectiveness of such measures in protecting the health of pregnant working women.

Future investigations should be planned to better define the molecular modes of action of different occupational risk factors. Physically demanding work may increase catecholamine levels [71,72,73,74] thus affecting constriction/dilation of blood vessels [75]. Indeed, high levels of catecholamines have been demonstrated in patients suffering from PE [75] and have been hypothesized to decrease uterine blood flow and influence early placentation [71]. An animal investigation performed on pregnant rats demonstrated that WBV exposure could result in increased plasma levels of plasma corticosterone and a decreased uterine flow [76], suggesting a possible role in the PE pathogenesis. However, all these aspects need additional deeper investigation.

Finally, some limitations characterize our revision that should be stressed in order to plan future methodologically adequate studies able to provide more informative data. First of all, the variability in the experimental designs should be considered, including different sample sizes and modes to assess the “exposure” experienced by the working women. In fact, the several types of jobs performed, and occupational risks experienced, evaluated through job titles, job exposure matrices as well as by quantitative exposure assessments in some studies, together with the not always available possibility to adjust for many confounders, including co-exposures in occupational settings, could have played a role in determining not homogeneous and difficult to compare results. Moreover, the choice to cover decades of literature in our review, in order to avoid losing just limited data, may have included studies addressing conditions of workplace exposure greatly changing over time and should be carefully considered to understand possible HDP–work relationships.

Generally, all the retrieved studies lacked an HDP diagnosis date. This prevented the authors from controlling for the number of days away from work (sick leave, parental leave or pregnancy allowance), and from understanding whether the absence was due to the HDP outcome or not. Finally, occupational exposures can be complex, heterogenous and difficult to define and characterize. Indeed, misclassification of workers was possible. Some exposure misclassification could be also due to the absence of information regarding reassignment or changes in tasks that could occur at some point in the pregnancy to reduce certain types of exposure. Some limitations in the studies may also regard the dichotomous classification of the exposure as present or not present, therefore preventing appropriate categorization in subgroups. In addition, information about working full or part time, which decreases the risk of underestimating the association due to dilution of the associations by the part-time workers were rarely available. Furthermore, the timing of exposures during pregnancy, that can be key to determining the type and severity of maternal and fetal adverse health effects, was rarely provided.

The healthy worker effect, a bias that results because workers tend to be better fit both physically and psychologically than the unemployed population, can characterize another limitation to consider for a correct interpretation of the results. This type of bias potentially produces an underestimation of the association between occupational stress and disease. Furthermore, a correct interpretation of available data is even more complicated by the intra- and inter-individual differences among women, in terms of age, body mass index, parity, educational levels that can all function as potentially HDP affecting variables. Finally, history of hypertension or PE was considered among the exclusion criteria in most of the revised studies. However, this aspect should be deeply evaluated from an occupational health perspective, as women with such anamnesis should deserve targeted and possibly stricter preventive measures and personal plans of occupational medical surveillance to avoid a further increase HDP risk.

## 5. Conclusions

In conclusion, although the association between occupational risk factors and HDP has been studied for decades, definite information is not yet available. Future investigations should be focused on defining the possible role of different occupational risk factors, the influence of co-exposures and the relationship with individual and personal issues in determining the risk for HDP development and evolution in pregnant workers. Overall, from an occupational and public health approach, this may be important to define suitable strategies for targeted risk assessment and management measures in different occupational settings aimed at protecting the health of pregnant women and their children.

## Figures and Tables

**Table 1:** Studies assessing the relationship between hypertensive disorders in pregnancy (HDP), including preeclampsia (PE) and pregnancy induced hypertension (GH), and employment status.

Study Location (Analyzed Period)	Study Design	Population Investigated (Number) and Age	Occupational Risk Factors	Additional Information	Results	Overall Quality	Reference
Pennsylvania and New York, USA (1987–1989)	Prospective cohort	717 women with singleton pregnancies	Employment status	*-*	Higher risk of GH: All cases among the group who worked during first semester vs. no cases in the unemployed group (lower bound of 95% CI for RR = 1.7).	Good	Landsbergis et al. [29]
North Carolina, USA (1984–1987)	Cross sectional	110 cases of PE vs. 115 controls	Employment status	*-*	Higher risk of PE: ✓ In working women compared to unworking ones (OR 2.3; 95% CI 1.2–4.6).	Good	Klonoff-Cohen et al. [30]
Dublin, Ireland (not specified)	Prospective cohort	933 primiparas with singleton pregnancies 289 unemployed vs. 245 employed	Employment status	*-*	Higher risk of PE: ✓ Working women vs. not working (aOR 5.48, 95% CI 1.08–27.76).	Good	Higgins et al. [31]
Netherlands (2002–2006)	Prospective cohort	4465 women with singleton pregnancies	Employment status Working hours	Working hours per week: 1–24; 25–39: 40 or more.	Higher risk of GH and PE, although not statistically significant: ✓ Women who quit their job before 34 weeks of gestation vs. those who do not.	Excellent	Nugteren et al. [32]
Iowa, USA (2002–2005)	Cross sectional	258 primiparous women with PE and 233 primiparous women with GH vs. 182 primiparous normotensive women	Employment status Working hours	Women were asked to report whether or not they worked during pregnancy and the number of hours worked per week.	Lower risk of PE: ✓ More than 8.25 h per day (aOR 0.58, 95% CI 0.36–0.95) vs. being active less than 4.2 h per day.	Good	Spracklen et al. [33]
Iceland (2005–2012)	Retrospective cohort	35,211 women with singleton pregnancies	Employment status	Employment status: ✓ Employed; ✓ Student; Not working (homemaker/unemployed/on disability).	Unemployment led to higher risk of: ✓ HDP (aOR 1.03, 95% CI 1.00–1.06) with each percentage point increase in unemployment (aOR 1.08, 95% CI 1.04–1.13) with each percentage point increase in unemployment. ✓ There was no association with PE.	Good	Eiríksdóttir et al. [34]
Canada (1997–1999)	Retrospective cohort	4729 women who delivered singleton births and worked since the first month of pregnancy	Employment status Work schedule	Information on work conditions at the onset of pregnancy was collected.	Higher risk of PE ✓ Working more than 5 consecutive days (3.0 aOR, 95% CI 1.0–9.5) vs. working less than 5 consecutive days.	Good	Haelterman et al. [35]
Connecticut, USA (1988–1991)	Cross sectional	142 PE and 172 GH vs. 2422 normotensive controls	Employment status Work activity	-	Lower risk of PE, although non-significant: ✓ Unemployed (aOR 0.64, 95% CI 0.21–2.00) vs. highest sitting engaged workers. No indication of a protective effect for GH of unemployment.	Excellent	Saftlas et al. [36]
Taiwan (2005)	Retrospective cohort	20,276 primiparous women with singleton pregnancies	Type of employment	Work schedules subgroups: ✓ Daytime only; ✓ Evening only; ✓ Daytime and evening only; ✓ Rotating shift. Working hours subgroups: ✓ ≤40; 41–48; 49–56; >56 h/week.	No significant findings between non-employed and different work schedules or working hours in all or primiparous women.	Good	Chang et al. [37]
Texas, USA (2005)	Retrospective cohort	385,537 women who gave birth	Type of work Education	Study limitations: Data obtained by the Texas Electronic Registrar Birth Registration, with self-reported information.	Higher risk of HDP: ✓ Non-homemakers (aOR 1.13, 95% CI 1.09–1.17) vs. homemakers. Within nonhomemakers: ✓ Student or unemployed (aOR 1.31, 95% CI 1.22–1.41) vs. homemakers; ✓ Sales (aOR 1.09, 95% CI 1.01–1.17) vs. homemakers; ✓ Educational Services (aOR 1.18, 95% CI 1.09–1.26) vs. homemakers; ✓ Business and Management (aOR 1.14, 95% CI 1.08–1.20) vs. homemakers; ✓ Healthcare (aOR 1.58, 95% CI 1.13–1.28) vs. homemakers; ✓ Legal and Social services (aOR 1.80, 95% CI 1.10–1.37) vs. homemakers. Prepregnant overweight/obesity increases the risk within the categories of job at risk.	Good	Bilhartz et al. [38]
Italy (1990–1994)	Retrospective cohort	160 women with severe PE vs. 320 normotensive women	Work type	-	Lower risk of PE: Clerical workers (OR 0.53, 95% CI 0.30–0.96) vs. unemployed women; Clerical workers (OR 0.2 95% CI 0.08–0.049) vs. not formally employed women.	Good	Spinillo et al. [39]
Mexico City, Mexico (1992)	Retrospective cohort	2663 women with singleton pregnancies	Socio-economic status Work type Education	Socio-economic status: ✓ Uninsured (SSA); ✓ Private sector employees (IMSS); ✓ Public sector employees (ISSSTE).	Higher risk of PE: ✓ SSA (OR 3.1, 95% CI 2.2–4.5) vs. IMSS ✓ <4 years of education (OR 4.1, 95% CI 2.0–8.6) vs. >12 years of education ✓ >4 years of education: work in service (OR 1.68., 1.01–2.81) or retail (OR 1.99, 95% CI 1.18–3.37) vs. other occupations ✓ No permanent job (OR 2.7, 95% CI 1.9–3.9) vs. permanent job ✓ Primiparas (OR 2.64, 95% CI 1.65–4.21) vs. 1–2 parity	Good	Cerón-Mireles et al. [40]

**Table 2:** Studies assessing the relationship between hypertensive disorders in pregnancy (HDP), including preeclampsia (PE) and gestational hypertension (GH), and exposure to chemical risk factors.

Study Location (Analyzed Period)	Study Design	Population Investigated	Occupational Chemical Agents	Additional Information	Results	Overall Quality	Reference
Netherlands (2002–2006)	Prospective cohort	4465 women with singleton pregnancies	Occupational chemical compounds: polycyclic aromatic hydrocarbons (PAHs); pesticides; phthalates; organic solvents; bisphenol A; alkylphenolic compounds; flame retardants; Metals; miscellaneous agents	Job Exposure Matrix (JEM) scores: ✓ Exposure unlikely; ✓ Exposure possible; ✓ Exposure probable.	Higher risk of PE in women exposed to pesticides, although not statistically significant (3.15, 95% CI 0.38–25.94) vs. no exposure.	Excellent	Nugteren et al. [32]
New Haven, Connecticut, North America (1980–1982)	Prospective cohort	270 women who delivered at Yale-New Haven Hospital: 90 occupational exposed vs. 180 controls	Solvents	Job Titles: Laboratory workers/technicians (n = 22); artist/art teachers (n = 16); assemblers (n = 13); machine operators/factory workers (n = 5); operating and recovery room personnel (n = 11); electrical and circuit-board workers (n = 5); science teachers (n = 3); chemist/chemical workers (n = 5); layout editors (n = 2).	Higher risk of: ✓ PE (aRR 3.9, 95% CI 2.5–5.4) exposed vs. not exposed; ✓ GH (aRR 3.0, 95% CI 0.9–9.9), exposed vs. not exposed, although not significant.	Good	Eskenazi et al. [45]
Sicily, Italy (2007–2013)	Prospective cohort	2203 women who delivered during the period. 474 occupational exposed vs. 582 not exposed	Organophosphorus pesticides: diazinon; malathion; parathion; chlorpyrifos	Exposure in the first trimester of pregnancy: ✓ No exposure; ✓ Indirect exposure; ✓ Domestic exposure; ✓ Occupational exposure.	Higher risk of GH: Exposure to diazinon (aOR 1.09, 95% CI 1.03–1.16) and malathion (aOR 1.14, 95% CI 1.08–1.19) vs. no exposure.	Sufficient	Ledda et al. [46]
U.S. Navy Personnel (1987–1989)	Retrospective cohort	5605 women with singleton pregnancies	Hazardous chemical exposure (e.g., organic, metals, etc.)	Exposure to chemical agents classified as: ✓ Never exposed during workday); ✓ <50% of the work day; ✓ ≥50% of the work day.	No increased risk of HDP due to hazardous chemical exposure in the workplace.	Good	Irwin et al. [47]

**Table 3:** Studies assessing the relationship between hypertensive disorders in pregnancy (HDP), including preeclampsia (PE) and gestational hypertension (GH), and exposure to physical risk factors.

Study Location (Analyzed Period)	Study Design	Population Investigated	Occupational Risk Factors	Additional Information	Results	Overall Quality	Reference
Canada (1997–1999)	Retrospective cohort	4729 women who delivered singleton births and worked since the first month of pregnancy	WBV Extreme temperatures Noise	WBV: Any exposure Extreme temperatures: always or frequently; rarely or never. Noise: an environment where a person could speak normally (not exposed); or had to speak loudly or shout (exposed) to be heard at 2 m.	WBV: no significantly higher risk of PE in exposed vs. unexposed women (1.2 aOR, 95% CI, 0.6–2.5). Extreme temperatures: higher risk of PE (1.6 aOR, 95% CI, 1.0- 2.6) and GH (1.3 aOR, 95% CI, 0.8–2.2), in women frequently or always exposed to extreme temperatures vs. those rarely or never exposed. Noise: no significant association.	Good	Haelterman et al. [35]
U.S. Navy Personnel (1987–1989)	Retrospective cohort	5605 women with singleton pregnancies	Noise Extreme temperature/humidity	Intensity and duration of noise exposure during a typical working day: High: ≥84 dB(A)/8 h. Medium: >conversation levels. Low: rarely or never > conversation levels.	No association with noise exposure No association with extremes temperatures and humidity.	Good	Irwin et al. [47]
Sweden (1994–2014)	Prospective cohort	1,109,516 working women with singleton pregnancies	Noise	Annual average 8 h occupational exposure levels: 70 dB(A); 70–74 dB(A); 75–80 dB(A); 80–85 dB(A); >85 dB(A).	Noise exposure 80–85 dB(A) both in part-time and full-time employment: Higher risk of HDP (aRR 1.10, 95% CI 1.06–1.44) and PE (aRR 1.11, 95% CI 1.07–1.16) vs. <70 dB(A). Noise exposure 80–85 dB(A) only in full-time employment: Higher risk of HDP (aRR 1.12, 95% CI 1.05–1.18) and PE (aRR 1.14, 95% CI 1.07–1.22) vs. <70 dB(A). No significant association for exposure >85 db(A).	Good	Lissaker et al. [50]
Norway (October–November 1989)	Retrospective cohort	5388 women with singleton pregnancies	Noise	Any exposure.	No association.	Insufficient	Wergeland and Strand. [51]
Finland (1976–1985)	Case–control	1475 mothers from the Finnish Register of Congenital Malformations between June 1976 and December 1985 vs. 1475 controls	Noise and night shift work	Any exposure. No exposure (less than 80 dB(A)). Low intensity (around 80 dB(A)). Moderate (around 85 dB(A)). High intensity (around 90 dB(A)).	Higher risk of GH in exposed mothers compared to unexposed (RR 1.8, 95% CI 1.0–1.3).	Sufficient	Nurminenand Kurppa [52]
Sweden (1994–2014)	Prospective cohort	1,091,044 working women who gave birth between 1994 and 2014	Whole Body Vibrations (WBV)	Average 8 h occupational exposure levels: High: ≥0.5 m/s^2^. Medium: 0.3–0.4 m/s^2^. Low: 0.1–0.2 m/s^2^.	Higher risk for full-time workers exposed to WBV at levels ≥0.5m/s^2^: ✓ PE (aOR 1.76, 95% CI 1.41 to 2.20) vs. not exposed; ✓ GH (aOR 1.55, 95% CI 1.26 to 1.91) vs. not exposed.	Good	Skröder et al. [53]

**Table 4:** Studies assessing the relationship between hypertensive disorders in pregnancy (HDP), including preeclampsia (PE) and gestational hypertension (GH), and exposure to physical workload.

Study Location (Analyzed Period)	Study Design	Population Investigated	Occupational Risk Factors	Additional Information	Results	Overall Quality	Reference
Pennsylvania and New York, USA (1987–1989	Prospective cohort	717 women with singleton pregnancies	First trimester physical work demands	Assessing Physical Workload score (range 0–980): sum of subjects’ responses to five items (climbing or balancing; lifting, carrying, pulling, or pushing objects; moving around a lot; exerting a lot of physical effort; sitting or standing in uncomfortable positions for long periods of time).	Physical work demands > 200: Lower risk of GH and PE although not statically significant (0.7 OR, 95% CI 0.2–2.5) vs. physical work demands < 200.	Good	Landsbergis et al. [29]
Ireland	Prospective cohort	933 primiparas with singleton pregnancies 289 unemployed vs. 245 employed	Employment Work posture	Work type defined as: ✓ Sedentary (n = 135) ✓ Standing (n = 50) ✓ Active (n = 49)	Higher risk of PE: ✓ Working women vs. not working (aOR 5.48, 95% CI 1.08–27.76). Higher blood pressure levels: ✓ Systolic and diastolic pressure in working vs. not working (*p* < 0.01); ✓ Night-time and 24 h diastolic pressure in active group vs. sedentary group (*p* < 0.05) There was no association with GH.	Good	Higgins et al. [31]
Netherlands (2002–2006)	Prospective cohort	4465 women with singleton pregnancies	Physical exertion	Occupational physical exertion: ✓ Long period of standing ✓ Long periods of walking ✓ Long periods of driving ✓ Manually handling loads ≥ 25 kg Working hours per week: 1–24; 25–39: 40 or more	Higher risk of GH and PE, although not statistically significant: ✓ Women who quit their job before 34 weeks of gestation vs. those who do not; No association with physically demanding work.	Excellent	Nugteren et al. [32]
Iowa, USA (2002–2005)	Cross selection	258 primiparous women with PE and 233 primiparous women with GH vs. 182 primiparous normotensive women	Leisure Time Physical Activity (LTPA) Work schedule Occupational physical exertion Work posture	Occupational risk factor investigated: ✓ Occupational work during pregnancy; ✓ Hours per week spent ✓ Carry/lift > 20 pounds ✓ Time spent on feet ✓ Time spent standing in one place ✓ Time spent sitting	Lower risk of PE: ✓ More than 8.25 h per day (aOR 0.58, 95% CI 0.36–0.95) vs. being active less than 4.2 h per day; ✓ One-hour increase in the amount of time being on feet at work (aOR 0.92, 95% CI 0.81–1.05).	Good	Spracklen et al. [33]
Canada (1997–1999)	Retrospective cohort	4729 women who delivered singleton births and worked since the first month of pregnancy	Work posture Physical exertion	Work posture assessed as hours spent: ✓ Walking; ✓ At the same place, without walking; ✓ Squatting or kneeling; ✓ With arms above shoulders levels; ✓ With back bent forward. Occupational physical exertion: ✓ Climbing stairs; ✓ Carrying or lifting loads; ✓ Pushing or pulling objects or persons.	Higher risk of PE: ✓ Standing at least 1 h without walking (aOR 2.5, 95% CI 1.4–4.6) vs. 0 h; ✓ Climbing stairs frequently (aOR 2.3, 95% CI 1.2–4.1) vs. never climbing stairs. Higher risk of GH: ✓ Pushing or pulling objects or persons > 5 times/day (aOR 1.9, 95% CI, 1.1–3.1) vs. never pushing or pulling objects or persons.	Good	Haelterman et al. [35]
Connecticut, USA (1988–1991)	Cross sectional	142 PE and 172 GH vs. 2422 normotensive controls	Work schedule Work posture Leisure-Time Physical Activity (LTPA)	✓ Sedentary vs. non sedentary groups; ✓ Least sitting vs. moderate sitting vs. highest sitting groups; ✓ LTPA intended as exercise or sport at least once per week for 12 months before pregnancy or during early pregnancy. Cross-stratified variable for yes/no LTPA and sedentary/non sedentary work.	Lower risk of PE, although non-significant: ✓ Regular LTPA (aOR 0.66, 95% CI 0.35–1.22) vs. no LTPA; ✓ Unemployed (aOR 0.64, 95% CI 0.21–2.00) vs. highest sitting; ✓ Non-sedentary jobs (aOR 0.71, 95% CI 0.37–1.36) vs. sedentary jobs; ✓ No indication of a protective effect of workplace activity. No indication of a protective effect for GH of workplace activity, LTPA or unemployment.	Excellent	Saftlas et al. [36]
Italy (1990–1994)	Retrospective cohort	160 women with severe PE vs. 320 normotensive women	Level of physical activity at work	Level of physical activity at work: ✓ No work: minimum activity; ✓ Mild: sedentary activity, rarely standing. ✓ Moderate: posture in standing position 20–30 h a week; ✓ High: standing or walking > 30 h a week.	Higher risk of PE: Moderate/high physical activity at work (OR 2.08, 95% CI 1.11–3.88) vs. mild activity.	Good	Spinillo et al. [39]
Mexico (1992)	Retrospective cohort	2663 women with singleton pregnancies	Work posture Physical activity at work	Hours spent standing and whether the job required physical effort assessed by structured questionnaire.	No association with hour spent standing, having a job which required physical effort.	Good	Cerón-Mireles et al. [40]
U.S. Navy Personnel (1987–1989)	Retrospective cohort	5605 women with singleton pregnancies	Physical job demands	Assessing Physical Workload: ✓ Standing; ✓ Lifting; ✓ Physical exertion.	Higher risk of PE in parous women engaged in the following activities vs. administrative and support jobs: ✓ Technical jobs (RR 6.5, 95%CI 1.7–25.3); ✓ Infantry seamanship position (RR 2.7, 95%CI 0.74–9.7); ✓ Unskilled labor (RR 2.6, 95%CI 0.82–7.5); ✓ Medium level of physical exertion (RR 2.5, 95%CI 1.2–5.0); ✓ Lifting (RR 2.0, 95%CI 0.87–4.5). Lower risk of PE in nulliparas women engaged in the following activities vs. administrative and support jobs: ✓ Craftsmen (RR 0.21); ✓ Unskilled labor (RR 0.44); ✓ High levels of lifting (RR 0.68, 95%CI 0.47–0.98).	Good	Irwin et al. [47]
Norway (October–November 1989)	Retrospective cohort	5388 women with singleton pregnancies	Work posture Physical job demands	Prolonged high physical work: ✓ Standing/walking; ✓ Standing with back bent forward; ✓ Twisting/bending. Occasional high physical work: working with hands above shoulders; lifting loads of 10–20 kg or >20 kg.	Higher risk of PE: ✓ Lifting heavy loads of 10–20 kg (aOR 1.8, 95%CI 1.2–2.5) vs. not lifting; ✓ Working with hands over shoulder level (aOR 1.4, 95%CI 1.0–2.2) vs. not working with hands over shoulder level.	Insufficient	Wergeland and Strand [51]

**Table 5:** Studies assessing the relationship between hypertensive disorders in pregnancy (HDP), including preeclampsia (PE) and gestational hypertension (GH), and shift work.

Study Location (Analyzed Period)	Study Design	Population Investigated	Occupational Risk Factors	Additional Information	Results	Overall Quality	Reference
Netherlands (2002–2006)	Prospective cohort	4465 women with singleton pregnancies	Work schedule	Night shift/month: ✓ Never; ✓ Occasionally; ✓ Often; ✓ Very Often.	No significant association.	Excellent	Nugteren et al. [32]
Canada (1997–1999)	Retrospective cohort	4729 women who delivered singleton births and worked since the first month of pregnancy	Work schedule	Weekly night work hours (23:00–06:00): ✓ None; ✓ ≥1.	No significant association.	Good	Haelterman et al. [35]
Taiwan (2005)	Retrospective cohort	20,276 primiparous women with singleton pregnancies	Work schedule Type of employment	Work schedules subgroups: ✓ Daytime only; ✓ Evening only; ✓ Daytime and evening only; ✓ Rotating shift. Working hours subgroups: ≤40; 41–48; 49–56; >56 h/week.	No significant findings between non employed and different work schedules or working hours in all or primiparous women.	Good	Chang et al. [37]
Norway (October–November 1989)	Retrospective cohort	5388 women with singleton pregnancies	Work schedule	Not specified.	Higher risk of PE only in parous women (aOR 2.0, 95%CI 1.1–3.6) vs. non-shift workers.	Insufficient	Wergeland and Strand [51]
Denmark (2007–2013)	Retrospective cohort	18,724 primiparous women with singleton pregnancies 11,193 night workers vs. 7531 day workers	Work schedule	Consecutive night shifts categories: ✓ 0: only single night shifts; ✓ 2–3: at least one spell of 2–3 consecutive night shifts and no spell of ≥4 consecutive night shifts ✓ ≥4: at least one spell of ≥4 consecutive night shifts.	Higher risk of HDP: ✓ Women working ≥ 4 night shifts (aOR 1.41, 95%CI 1.01–1.98) vs. 0 night shifts; ✓ Positive trend for consecutive night shifts (p for trend = 0.04); ✓ Women with BMI ≥ 30 working ≥ 4 night shifts (aOR 5.31, 95%CI 1.98–14.22) vs. women with BMI > 30 day workers. ✓ Women with BMI ≥30 without night shifts (aOR 3.47, 95% CI 1.15–10.52) vs. women day workers with BMI > 30.	Good	Hammer et al. [58]
Japan (2011–2014)	Prospective cohort	99,744 women with singleton pregnancies	Work schedule	Workers without night shifts: ✓ Working hours 1–35 h/w; ✓ Working hours 36–45 h/w; ✓ Working hours ≥46 h/w; Workers with night shifts: ✓ Same three categories for working hours, divided by two night shifts categories; ✓ Night shifts 1–5 d/m; ✓ Night shifts ≥6 d/m; Separate analyses were conducted for first and second/third trimester.	Higher risk of HDP: ✓ Women working ≥ 46 h/w with night shifts 1–5 d/m during second/third trimester (aOR 2.02, 95% CI 1.39–2.93) vs. non-workers; ✓ Women working 36–45 h/w with night shifts 1–5 d/m during second/third trimester (aOR 1.56, 95% CI 1.14–2.12) vs. non-workers; ✓ Women working 36–45 without night shifts (aOR 1.15, 95% CI 1.02–1.29) vs. non-workers. ✓ Women working ≥ 46 h/w without night shifts during first trimester (aOR 1.20, 95% CI 1.03–1.40) vs. non-workers.	Good	Suzumori et al. [59]
Iran (2017)	Retrospective cohort	429 working women with singleton pregnancies 214 shift workers vs. 215 day workers	Work schedule	Job title: ✓ Physician, nurse, nursing aid, operating room personnel (31.3%); ✓ Teacher (elementary teacher to university professor) (9%); ✓ Office workers and industrial workers (23.8%); ✓ Others (19.7%).	Higher risk of PE: Shift workers vs. morning workers (14.5% vs. 7.9%, *p* = 0.031). Non-significant higher risk of PE: Shift workers vs. morning workers (aOR 1.69, 95% CI 0.8–3.3).	Sufficient	Davari et al. [60]
Finland (1976–1985)	Case–control	1475 mothers from the Finnish Register of Congenital Malformations vs. 1475 controls	Rotating shifts in noisy and noiseless environment	✓ Any shift. ✓ Two shifts. ✓ Three shifts.	Higher risk for GH in shift work in a noisy environment compared to noiseless shift work (aRR 1.9, 95% CI 0.6–2.3)	Sufficient	Nurminen [61]

**Table 6:** Studies assessing the relationship between hypertensive disorders in pregnancy (HDP), including preeclampsia (PE) and gestational hypertension (GH), and exposure to job related stress.

Study Location (Analyzed Period)	Study Design	Population Investigated	Job Related Stress Variables	Additional Information	Results	Overall Quality	Reference
Pennsylvania and New York, USA (1987–1989)	Prospective cohort	717 women with singleton pregnancies	Working hours Job strain Job complexity	Job pressures/low job controls: ✓ Being pressured to get things done on time; ✓ Depending on other people’s schedules. Job decision latitude: ✓ Making a lot of decisions; ✓ Having high level of skill; ✓ Doing many different tasks; ✓ Being intellectually charged. Job complexity: ✓ High job decision latitude; ✓ Gaining cooperation of others; ✓ Talking a lot to others.	In women with lower-status jobs: ✓ Low decision latitude (SOR 2.4, 95% CI 1.1–5.2) vs. high decision latitude; ✓ Low job complexity (SOR 2.1, 95% CI 1.0, 4.6) vs. high job complexity. In women with high-status jobs: Job pressure/low control (SOR 3.6, 95% CI 0.9–15.1);	Good	Landsbergis et al. [29]
North Carolina, USA (1984–1987)	Cross sectional	110 cases of PE vs. 115 controls	Job strain	Job strain categories, by psychological demand and decision latitude: ✓ Low demand, high latitude; ✓ Low demand, low latitude; ✓ High demand, high latitude High demand, low latitude.	Higher risk of PE: ✓ High-strain jobs (aOR 3.1, 95% CI 1.2–7.8) vs. nonworkers; ✓ High-strain jobs (aOR 2.1, 95% CI 0.7–6.2) vs. all other workers; ✓ Low-strain jobs (aOR 2.0, 95% CI 1.0–4.3) vs. nonworkers; Workers (aOR 2.3, 95% CI 1.2–4.6) vs. nonworkers.	Good	Klonoff-Cohen et al. [30]
Canada (1997–1999)	Retrospective cohort	4729 women who delivered singleton births and worked since the first month of pregnancy	Job strain Work pace	Forced pace, piece work or assembly-line work Job strain: ✓ Low demand, high latitude; ✓ Low demand, low latitude; ✓ High demand, high latitude; High demand, low latitude.	No association to job strain and work pace.	Good	Haelterman et al. [35]
Norway (October–November 1989)	Retrospective cohort	5388 women with singleton pregnancies	Work pace	Work pace: ✓ Hectic work pace; ✓ Influence on work pace.	Higher risk of PE: Hectic work pace (aOR 1.4, 95% CI 1.0–2.0) vs. no hectic work pace. Lower risk of PE: Influence on work pace (aOR 0.7, 95% CI 1.0–2.0) vs. no influence.	Insufficient	Wergeland and Strand [51]
Quebec, Canada (1984–1986)	Cross sectional	128 PE cases, 201 GH cases vs. 401 normotensive women	Job strain Working hours	Job strain categories, by psychological demand and decision latitude: ✓ Low demand, high latitude; ✓ Low demand, low latitude; ✓ High demand, high latitude; ✓ High demand, low latitude. High job strain subgroups: ✓ <20 weeks spent on job vs. 20 weeks; ✓ <35 working hours per week vs. ≥35.	Higher risk of PE: ✓ High psychological demand (aOR 2.7, 95% CI 1.3–5.6) vs. low demand; ✓ High job strain (aOR 2.1; 95% CI 1.1–4.1) vs. low job strain. Higher risk of GH: High psychological demand (aOR 2.1, 95% CI 1.1–3.8) vs. low demand. High job strain (aOR 1.3; 95% CI 0.8–2.2) vs. low job strain.	Good	Marcoux et al. [63]
Lagos, Nigeria (2001–2002)	Case–control study	128 women who delivered during the period and who had PE Vs. 240 controls	Working during pregnancy Stressful work environment	Five-levels activity score based on: ✓ Distance of workplace from home. ✓ Type of transportation used to work; ✓ Type of work; ✓ Physical workload. ✓ Weekly working hours.	Higher risk of PE: Women with a stressful work environment during pregnancy (aOR 2.10; 95% CI 1.20–3.71) vs. unexposed controls. No association between working during pregnancy and PE.	Sufficient	Anorlu et al. [64]
Amsterdam, Netherlands (2003–2004)	Prospective cohort	3679 primiparas with singleton pregnancies (128 PE, 161 GH, Vs 3390 controls)	Working Hours Workload Work control Job strain	Weekly working hours: <32, >32 h. Workload: Low: <50th percentile; Moderate: 50th–90th percentile; High > 90th percentile. Work control: High: >50th percentile; Moderate: 10th–50th percentile); Low: <10th percentile. Job strain: Highest: high workload, low work control; High: high workload, low-moderate work control; Low: low workload, high-moderate work control.	No significant association.	Sufficient	Vollebregt et al. [65]
Germany (2004–2006)	Cross sectional	725 cases vs. 880 controls	Emotional stress	Reasons for acute emotional stress, related to job: ✓ Unemployment; ✓ Occupational problems.	Causes for emotional stress: ✓ Occupational problems (p < 0.05); ✓ Unemployment (1.7% in patients vs. 0.6% in controls, non-significant).	Sufficient	Leeners et al. [66]
